# Effect of Slice Thickness Variations on Knee Cartilage Quantification Using Magnetic Resonance Image Compilation Sequence

**DOI:** 10.2174/0115734056427749260108092529

**Published:** 2026-02-02

**Authors:** Wenhao Wu, Yiyin Hu, Dantian Zhu, Wei Li, Wen Yu, Shaolin Li, Yucun Huang, Yijie Fang

**Affiliations:** 1 Department of Radiology, Fifth Affiliated Hospital of Sun Yat-sen University, Zhuhai, China; 2 Guangdong Provincial People's Hospital, Jinwan Central Hospital of Zhuhai, Zhuhai, China

**Keywords:** Knee joint cartilage, Quantitative techniques, Magnetic resonance image compilation, Slice thicknesses, T1-relaxation time, T2-relaxation time, PD values

## Abstract

**Introduction:**

This study aimed to evaluate the impact of varying slice thickness on quantitative values using the Magnetic Resonance Image Compilation (MAGiC) sequence.

**Methods:**

In this retrospective study, 23 healthy subjects underwent the MAGiC sequence (at 3.0 T) with three slice thicknesses: 3 mm (TH3), 4 mm (TH4), and 5 mm (TH5). The T1, T2, and PD values were measured in various knee joint cartilage regions by two experienced radiologists, including the lateral femoral condyle (LFC), lateral tibial plateau (LTP), medial femoral condyle (MFC), medial tibial plateau (MTP), patella (PAT), and trochlea (TRO). The effects of varying slice thicknesses (TH4 *vs*. TH3 and TH5 *vs*. TH3) were analyzed using paired t-tests or Wilcoxon signed rank tests, with statistical significance set at *P* < 0.025. Intra-rater and inter-rater reliability were also assessed.

**Results:**

Measurements of T1, T2, and PD values demonstrated high intra- and inter-rater reliability. Minimal differences were observed across slice thicknesses for T1WI, T2WI, and PDWI images. T2 and PD values showed little variation, while T1 mapping revealed significant differences. T2 values were consistent across regions, except for the LFC.

**Discussion:**

TH4 and TH5 can replace TH3 for knee joint scanning while reducing scan time, with minimal differences in anatomical depiction across sequences. MAGiC technology significantly improves efficiency by acquiring quantitative data in a single scan, demonstrating stable T2 values unaffected by slice thickness, though T1 and PD values are thickness-dependent. This technique holds clinical value for cartilage injury assessment but requires further research on the applicability of multiplanar imaging.

**Conclusion:**

T2 values obtained with the MAGiC sequence are stable across TH3, TH4, and TH5, allowing for reliable cartilage T2 quantification using TH5 to reduce patient scan time.

## 1 INTRODUCTION

Magnetic resonance imaging (MRI) provides high-resolution imaging of knee soft tissues, cartilage, and bone, effectively detecting subtle pathological changes such as cartilage defects, bone marrow edema, synovitis, and joint effusions, which are critical for diagnosing knee injuries [1-3]. Conventional MRI sequences, however, typically identify cartilage damage only after significant signal and morphological alterations have occurred, a stage often beyond spontaneous recovery. Comprehensive knee joint imaging necessitates multi-planar and multi-spectral sequences [4], which inherently require long acquisition times. In clinical practice, variations in knee joint morphology due to age, weight (*e.g*., obesity), or disease status frequently necessitate adjustments in slice thickness (TH) and interslice gap to optimize image quality [4]. Nevertheless, a narrower section thickness often prolongs scan duration, potentially reducing patient tolerance. Therefore, a key breakthrough sought by radiologists is to substantially reduce patient scan time without compromising image quality.

Quantitative MR techniques such as T1ρ [5], T2 mapping [6], and T2 * mapping [7] offer quantitative assessments of knee joint tissues, enabling earlier diagnosis and intervention [1, 8]. Despite rapid advancements [9-13], these quantitative techniques often entail longer scan times compared to conventional MRI and may raise specific absorption rate (SAR) concerns, potentially limiting their broad clinical applicability [14, 15]. The Magnetic Resonance Image Compilation (MAGiC) technology streamlines the conventional MRI workflow [16, 17]. Through a single Multi-Dynamic Multi-Echo Sequence (MDME) scan, it can reconstruct a wide array of contrast-weighted images, including Proton Density (PD) weighted image, fluid-attenuated inversion recovery (FLAIR), short-tau inversion recovery (STIR), phase-sensitive inversion recovery (PSIR), and double inversion recovery (DIR) [18, 19]. Furthermore, quantitative parameters of tissue biophysical properties (*e.g*., T1 and T2 relaxation times and their relaxation rates R1 and R2, PD values, *etc.*) can be directly obtained without additional scanning time [20-22]. The MAGiC sequence is relatively insensitive to the main magnetic field inhomogeneity, thereby reducing susceptibility artifacts [17]. Recent studies indicate that quantitative values obtained *via* the MAGiC technique across scanners from different manufacturers show a strong positive correlation with reference standards and exhibit low intra-group coefficients of variation, underscoring its accuracy and reproducibility [23]. The feasibility of MAGiC for musculoskeletal imaging has been increasingly validated [24-26]. For instance, Chen *et al.* recently demonstrated that the T1 and T2 values derived from MAGiC provide high diagnostic accuracy for osteoporosis [27].

However, the MAGiC composite image's quality depends on the scanning quality of a single MDME sequence [28]. Motion artifacts may arise during longer scans in patients over a longer period. Increasing the layer thickness to shorten the scanning time while maintaining the image quality can make MAGiC technology more advantageous for clinical applications [28]. Such an advancement is especially pertinent for conditions like knee osteoarthritis (KOA). Systematic reviews of KOA management, including those assessing therapeutic ultrasound [29], underscore the demand for efficient, quantitative imaging biomarkers. MAGiC-derived T1, T2, and PD values have the potential to serve as objective, sensitive measures of cartilage response to therapy, providing critical early feedback that conventional imaging cannot [28].

To this end, we systematically investigated how variations in slice thickness affect the quantification of T1, T2, and PD values from the MAGiC sequence. Our objective was to determine an optimal balance that minimizes scan time while preserving the reliability of quantitative measurements for clinical and research applications.

## 2 MATERIALS AND METHODS

The Institutional Review Board (IRB) approved this experiment. All procedures were performed in compliance with relevant laws and institutional guidelines and have been approved by the Ethics Committee. Each participant received a description of the planned technique alongside the study's goal, and they were required to sign written informed consent forms. A questionnaire was utilized to gather information on the subjects.

### 2.1 Subject Recruitment

This study was designed as a technical optimization and methodological investigation to evaluate the impact of slice thickness on quantitative cartilage parameters using the MAGiC sequence. To establish a baseline understanding without the confounding influence of disease, we recruited asymptomatic healthy volunteers. The inclusion and exclusion criteria are specified as follows: Inclusion criteria: Healthy volunteers who were able to complete MRI scans of both knees and provided written informed consent. Exclusion criteria:(1) Any history of knee pain, stiffness, previous knee trauma, acute knee injury, or orthopedic surgery involving the knee joint;(2) Presence of MRI contraindications (*e.g*., pacemaker implantation, metal clips, or other ferromagnetic implants);(3) Inability to cooperate with the scan protocol (*e.g*., due to claustrophobia). The volunteers underwent MR scans of both knee joints.

### 2.2 MRI Examination

This study is a prospective cohort study. We recruited a group of volunteers who met strict health criteria to form a research cohort. By performing repeated knee joint MAGiC sequence scans with three different slice thicknesses (3mm, 4mm, and 5mm) on all individuals within this cohort, we systematically evaluated the impact of changes in the technical parameter (slice thickness) on quantitative cartilage parameters (T1, T2, PD). MR examination was performed using a clinical 3-T MRI scanner (PIONEER, GE Healthcare) with an 8-channel coil. Sagittal images were acquired using the MAGiC sequence: flip angles = 120° [saturation], 90° [excitation], and 180° [refocusing] [30]. All subjects were scanned using three combinations of different slice thicknesses: 3 mm (TH3, scan time: 8 min 18 s), 4 mm (TH4, scan time: 6 min 18 s), and 5 mm (TH5, scan time: 5 min 36 s). The scanning range of the knee was standardized by adjusting the number of slices, which in turn triggered automatic adjustments to the repetition times (TRs) [30]. SpecificsequenceparametersaredetailedinTable **1**, with bilateral knee joints scanned separately.

### 2.3 Image Analysis

**Fig. (1):**
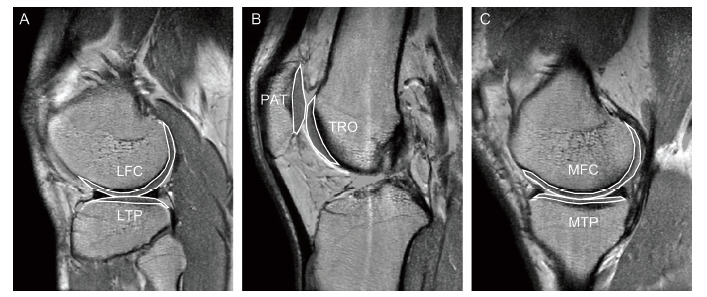
Diagram of the cartilage segmentation for data analysis. Regions of interest were placed on PD-weighted images. (**A**) Lateral cartilage compartment (LFC: lateral femoral condyle, LTP: lateral tibial plateau). (**B**) Patellofemoral cartilage compartment (PAT: patella; TRO: trochlea). (**C**) Medial cartilage compartment (MFC: medial femoral condyle, MTP: medial tibial plateau).

Knee cartilage quantification was performed by two musculoskeletal radiologists with more than five years of professional experience, who were blinded to participant details. ROIs were placed in a total of six knee cartilage regions: lateral femoral condyle (LFC), lateral tibial plateau (LTP), medial femoral condyle (MFC), medial tibial plateau (MTP), patella (PAT) and trochlea (TRO) [31] (Fig. **1**). Using ITK software (ITK-SNAP, Version 3, Copyright (C) 2007 Free Software Foundation, Inc. <http://fsf.org/>), the three consecutive levels with the best cartilage display in each subregion were selected on the T2 sagittal images, and the ROIs of each cartilage subregion was drawn manually. ROIs to obtain the T1, T2, and PD values of the cartilage in the corresponding regions, and calculate the mean value for each subregion. To ensure that both observers selected the same layers, the observers were trained before the drawing and the layers were recorded at the same time.

### 2.4 Intra- and Inter-rater Reliability Analysis

The intra-rater reliability (consistency of a single radiologist) and inter-rater reliability (consistency between the two radiologists) were assessed using a two-way mixed-effects intraclass correlation coefficient (ICC) in SPSS (Version 25.0, IBM). ICC values were interpreted as follows: < 0.50 (poor), ≥0.50 and < 0.75 (moderate), ≥0.75 and < 0.90 (substantial), and ≥0.90 (excellent consistency) [32].

### 2.5 Statistical Analysis

The Kolmogorov–Smirnov test confirmed that the T1, T2, and PD values all followed a normal distribution. For each subregion of the cartilage, the mean and standard deviation (SD) of T1, T2, and PD values were calculated. The influence of varying slice thicknesses on T1, T2, and PD values was analyzed using repeated-measures analysis of variance (ANOVA). When data distributions were normal (with a *P* value > 0.05), the paired t-test was applied to examine the effects of different slice thicknesses (comparing TH4 with TH3 and TH5 with TH3). For non-normally distributed data (*P* value ≤ 0.05), the paired Wilcoxon signed rank test was utilized. After Bonferroni correction, the significance threshold for both the paired t-test and the Wilcoxon signed rank test was adjusted to *P* < 0.025. It was recognized that an alternative, and more stringent, statistical viewpoint is to consider the entirety of the study's comparisons (6 regions × 3 parameters) as a single family. To address this, a post-hoc analysis was undertaken, which implemented a more stringent Bonferroni correction for all 18 comparisons, employing a significance threshold of *P* < 0.0014. All paired t-tests and Wilcoxon signed rank tests were performed using GraphPad Prism (version 8.0.0, Dotmatics).

## 3 RESULTS

The study included a total of 23 people (20 men and 3 women) who met the eligibility criteria to form a research cohort between October 2019 and March 2020. Volunteers were 28 to 50 (40.4 ± 6.0) years old, 155.0-185.0 (173.3 ± 7.3) cm in height, and 48.0-81.0 (66.4 ± 8.7) kg in weight. The volunteers underwent MR scans of both knee joints. After excluding images with poor SNR (mostly motion artifacts), a total of three groups of images of 41 knee joints were included for comparative analysis.

Table **2** presents the intra- and inter-rater reliability outcomes for cartilage assessments. Intra-rater reliability analyses revealed excellent consistency, with ICC values of 0.924 (95%CI: 0.915–0.933) for T1, 0.941 (95%CI: 0.933–0.947) for T2, and 0.976 (95%CI: 0.973–0.979) for PD. For inter-rater reliability, comparisons between the two experienced radiologists demonstrated substantial agreement, with ICC values of 0.869 (95%CI: 0.852–0.883) for T1, 0.909 (95%CI: 0.898–0.919) for T2, and 0.961 (95%CI: 0.956–0.965) for PD.

**Fig. (2):**
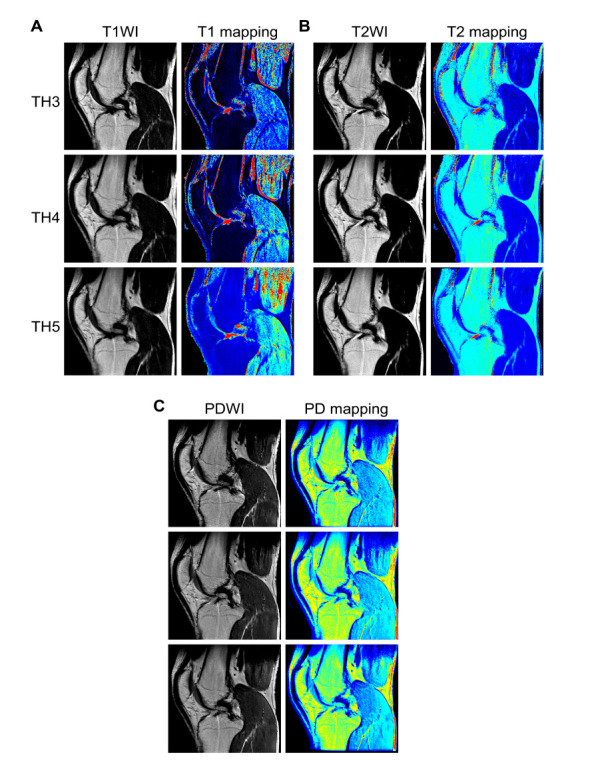
(**A**-**C**) Representative T1WI, T2WI, PAWI, and their corresponding mapping images from a subject for the patellofemoral cartilage compartment. TH3, slice thicknesses (mm). TH4, slice thicknesses (mm). TH5, slice thicknesses (mm).

Figures (**2****A**-**C**) showed representative T1, T2, and PD maps of the knee joint from a subject. The high SNR and contrast in the quantitative maps are clearly shown for all of the slice thicknesses. From the images of T1WI, T2WI, and PDWI, there was little difference in the scans of different slice thicknesses. Changes in the volume of cartilage at the same ROI on different slice thickness images were apparently negligible.In the T2 mapping(Fig. **2B**)andPDmapping(Fig. **2C**) images utilized for quantification, there was minimal difference observed between different slice thicknesses. Conversely, notable discrepancies were evident in the T1 mapping images across different slice thicknesses (Fig. **2A**).

Table **3** summarizes the T1, T2, and PD values for the six knee cartilage regions for the three slice groups. Data distributions of all cartilage regions except the T1 value of MTP were normal (*P* > 0.05, Kolmogorov–Smirnov test). The *P*-values calculated from the paired samples t-tests or the Wilcoxon signed rank test between the slice groups are summarized in Table **4**.

For all knee cartilage regions, T2 did not differ between any of the slice groups (*P* > 0.025) except the LFC. Statistically, although we applied a correction for the slice thickness comparisons (*P* < 0.025), a global correction accounting for all regions and parameters was not implemented in the primary analysis. As a supplementary measure, we performed a more conservative post-hoc analysis (global correction threshold of *P* < 0.0014). The results indicated that a statistically significant difference (*P* < 0.0014) was observed only between TH3 and TH4 in the lateral femoral condyle (LFC). However, the use of different slice thicknesses influencedtheT1valuesforvariouscartilageregions(Table **4**). As the slice thickness of the quantitative sequence increased, the T1 values progressively decreased. These differences were statistically significant according to both statistical analyses (Table **4**). The histogram plots show the mean and SD of the T2 relaxation times in each subregion of knee cartilage in Fig. (**3**).

In addition, the cartilage PD values in the MTP and TRO areas did not change much in different slice thicknesses. In other areas, the PD value increases with increasing slice thickness. In LTP and MFC, the PD values obtained from images of TH 5 and TH 4 were significantly different from those of TH 3. In LFC and PAT, only the PD values obtained in the images of TH 4 were not statistically different from the values of TH 3, while the values of TH 5 and TH 3 were significantly different (Table **4**).

## 4 DISCUSSION

Our research indicates that morphological observations of the patient's knee joint region suggest TH4 and TH5 can be used interchangeably with TH3 for scanning purposes. Additionally, to reduce scanning time, T2 values of the cartilage can be quantified using either TH5 or TH4 instead of TH3.

The results demonstrate that the final T1WI, T2WI, and PDWI images, in terms of depicting anatomic structures, exhibit minimal differences between TH4/TH5 and TH3. Compared to conventional relaxation quantification sequences that require multiple sequences, Zheng *et al.* found that MDME sequences were also robust, even varied in a range of scanning parameters (including ETL, matrix, and acceleration factor within the range of routine clinical use) [33]. MAGiC significantly reduces scan duration while maintaining consistent scanning conditions and standards, facilitating precise comparisons [16, 22, 34, 35]. It offers a one-stop process for quantitatively assessing knee structural abnormalities and cartilage injuries [20, 36]. Currently, MAGiC technology prioritizes reducing the total scanning time of multiple sequences in conventional MRI by increasing the scanning time of a single sequence. Consequently, the image quality of MAGiC composites heavily relies on the scanning quality of a single MDME sequence [28]. For patients unable to complete extended scans, motion artifacts may arise during longer scans [37, 38]. Therefore, increasing the layer thickness to shorten the scanning time while maintaining image quality can enhance the clinical applicability of MAGiC technology.

**Fig. (3):**
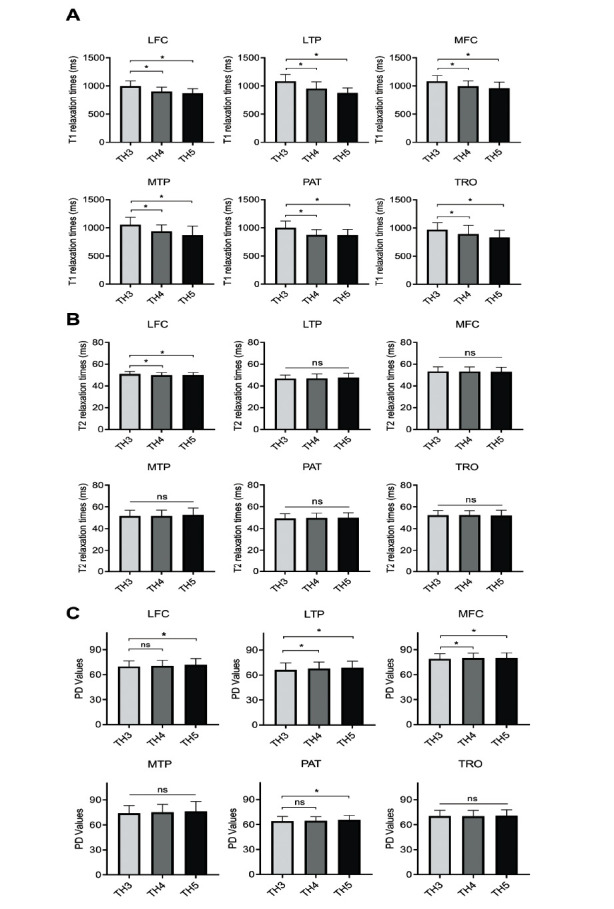
The histogram plots show the mean and SD of the T1 relaxation times (**A**), T2 relaxation times (**B**), and PD values (**C**) in each subregion of knee cartilage. The T2 values were robust regardless of the slice thicknesses. However, the slice thickness does affect the measurement of T1 and PD values. TH3, slice thicknesses (mm). TH4, slice thicknesses (mm). TH5, slice thicknesses (mm). LFC: lateral femoral condyle, LTP: lateral tibial plateau, MFC: medial femoral condyle, MTP: medial tibial plateau, PAT: patella, TRO: trochlea.

In this study, when quantitative values of knee cartilage were measured using various quantitative maps obtained by the MAGiC technique, we found that different slice thicknesses had little effect on the quantitative values obtained from T2 maps of most cartilage regions (except LFC) (Tables **3** and **4**, Fig. **3**). In a previous study using MDME sequences for brain scanning, T2 mapping showed the same robustness in measurements of different layer thicknesses [30].T2 mapping can numerically reflect early physiological changes in cartilage microstructure, including collagen matrix, fixed charge density (FCD), and water content, during cartilage degeneration [30]. T2 mapping provides crucial imaging evidence and supports clinical diagnosis and treatment. Considering the thinner cartilage in the LFC of the knee cartilage, a partial volume effect may account for the different T2 values when scanning with thicker slice thicknesses [39]. Although the T2 values were robust and remained consistent regardless of the slice thicknesses, we observed that the slice thickness does affect the measurement of T1 and PD values. This aligns with previous findings, which indicate that the calculation of tissue T1 values using the MAGiC technique varies with the thickness of the scanned layer, thereby influencing the accurate quantification of tissue T1 values [30].

Our findings regarding the impact of slice thickness on cartilage quantification have direct and important implications for clinical practice, particularly in the management of osteoarthritis (OA). The MAGiC sequence employed in this study is exceptionally well-suited for OA patients due to its unique ability to provide a comprehensive quantitative assessment of cartilage (including T1, T2, and PD values) from a single, time-efficient acquisition [25]. There is a pressing need to reduce scan times in busy clinical workflows to improve patient comfort and throughput. By optimizing slice thickness protocols as investigated here, clinicians can achieve accurate cartilage thickness and composition measurements with a scan time significantly shorter than that required by conventional multi-sequence MRI protocols. This makes our approach a promising tool for the longitudinal monitoring of disease progression or treatment response in OA, where repeated scans are necessary, and efficiency is paramount.

Our findings set the stage for deploying the optimized MAGiC protocol in large-scale cohorts to define normative values and monitor cartilage degeneration across populations [40]. The integration of artificial intelligence, a key trend in smart healthcare, offers a transformative next step [41]. Unlocking these data with deep learning could automate segmentation and quantification, revolutionizing assessment through high-throughput, reproducible analysis, and seamless clinical integration.

## 5 STUDY LIMITATIONS

The present study has the following limitations. Firstly, it solely employed the MAGiC technique for quantitative measurement of knee joint cartilage. To gain a more holistic understanding of the knee joint's overall imaging, it would be beneficial to simultaneously examine tissues such as knee ligaments and menisci. Secondly, our investigation focused exclusively on the impact of different slice thicknesses on quantitative measurements using the MAGiC sequence in sagittal images. To broaden its clinical applications, further comparative studies on corresponding slice thicknesses in coronal and axial images are recommended. Thirdly, the sample size of 23 healthy volunteers is relatively modest, and the final analysis included only 41 knee joints after the exclusion of images with poor quality, which may affect the generalizability of our findings for establishing normative values. The exclusion of images primarily due to motion artifacts, while necessary for data quality, highlights a challenge for clinical reproducibility. Future studies should incorporate strategies like motion-correction techniques to enhance the robustness of the method in routine practice. Furthermore, the study population was limited to healthy volunteers within a relatively narrow age range (28–50 years) with an unbalanced gender distribution (20 males and 3 females), which restricts the generalizability of the results to clinical populations—such as patients with osteoarthritis or post-traumatic knee conditions—where cartilage properties have undergone alterations. Future studies should recruit larger cohorts with more diverse clinical characteristics, balanced gender representation, and a broader age range, and extend the analysis to the coronal and axial planes to comprehensively validate and generalize the findings of this study.

## CONCLUSION

MAGiC sequences offer significant advantages in knee imaging by acquiring high-resolution soft tissue images while non-invasively obtaining quantitative tissue information. In this study, we explored the effect of utilizing MAGiC sequences with varying scanning slice thicknesses on knee scans. Our findings revealed that T2 values estimated using MAGiC sequences remained stable, regardless of the slice thickness employed. Therefore, the optimized MAGiC protocol shows great potential for efficient and accurate longitudinal monitoring of cartilage in osteoarthritis patients within routine clinical practice.

## Figures and Tables

**Table 1:** Multi-dynamic, multi-echo sequence parameters.

	TH3	TH4	TH5
TR (ms)	5931	4498	4000
TE (ms)	22.6/101.9	22.4/100.6	23.1/103.8
Number of sections	30	23	20
Interslice gaps (mm)	0	0	0
TI (ms)	Automatically calculated 4 different TI		
FOV (mm^2^)	150 × 150	150 × 150	150 × 150
Scan matrix	320 × 256	320 × 256	320 × 256
Asset factor	2	2	2
Echo-train length	14	14	14
Bandwidth (kHz)	25	25	25
NEX	1	1	1
Scan time	8 min 18 s	6 min 18 s	5 min 36 s

**Note:** TH, slice thicknesses (mm); TR, repetition time; TE, echo time; G, interslice gaps (mm); TI, inversion delay; FOV: field of view; NEX, the number of excitations.

**Table 2:** ICC values for intra- and inter-rater reliability analysis of knee cartilage quantification using the MAGiC sequence.

		Intra-rater Reliability	Inter-rater Reliability
T1	bulk	0.924 (0.915 – 0.933)	0.869 (0.852 – 0.883)
	TH3	0.883 (0.857 – 0.904)	0.866 (0.835 – 0.890)
	TH4	0.911 (0.892 – 0.928)	0.767 (0.715 – 0.810)
	TH5	0.940 (0.927 – 0.951)	0.897 (0.874 – 0.916)
T2	bulk	0.941 (0.933 – 0.947)	0.909 (0.898 – 0.919)
	TH3	0.930 (0.914 – 0.943)	0.908 (0.887 – 0.925)
	TH4	0.950 (0.939 – 0.959)	0.915 (0.895 – 0.930)
	TH5	0.943 (0.930 – 0.953)	0.904 (0.883 – 0.922)
PD	bulk	0.976 (0.973 – 0.979)	0.961 (0.956 – 0.965)
	TH3	0.968 (0.961 – 0.974)	0.950 (0.938 – 0.959)
	TH4	0.979 (0.974 – 0.983)	0.967 (0.959 – 0.973)
	TH5	0.983 (0.979 – 0.986)	0.965 (0.957 – 0.971)

**Note:** TH, slice thicknesses (mm); ICC < 0.50 for poor agreement, ≥ 0.50 and < 0.75 for moderate agreement, ≥ 0.75 and < 0.90 for significant agreement, and ≥ 0.90 for excellent agreement. The values in brackets indicate 95% confidence intervals (CI).

**Table 3:** T1, T2 and PD value for the three slice thicknesses.

	T1				T2				PD				
	TH3	TH4	TH5	*P* value	TH3	TH4	TH5	*P* value	TH3	TH4	TH5	*P* value
LFC	995.2 ± 95.5	898.0 ± 79.9	870.3 ± 82.3	<0.0001*	50.9 ± 2.5	49.9 ± 2.4	49.9 ± 2.4	0.0007*	69.8 ± 6.6	70.6 ± 6.5	71.9 ± 7.5	0.0003*
LTP	1084.0 ± 122.7	952.8 ± 122.5	873.2 ± 92.1	<0.0001*	46.8 ± 3.2	46.9 ± 4.0	47.8 ± 3.9	0.1343	66.2 ± 8.3	67.6 ± 7.8	68.5 ± 8.0	<0.0001*
MFC	1084 ± 99.8	993.5 ± 98.1	959.5 ± 109.9	<0.0001*	53.3 ± 4.2	53.3 ± 4.2	53.1 ± 4.0	0.7814	79.0 ± 6.0	80.0 ± 5.7	80.1 ± 6.3	0.0442*
MTP	1052.0 ± 133.2	936.8 ± 113.4	870.7 ± 157.7	<0.0001*	51.6 ± 5.3	51.7 ± 5.4	52.6 ± 6.3	0.2087	74.2 ± 9.1	75.2 ± 9.5	76.1 ± 11.8	0.0783
PAT	1002.0 ± 120.0	873.0 ± 96.5	971.8 ± 100.8	<0.0001*	49.3 ± 4.2	49.75 ± 4.4	49.9 ± 4.7	0.3265	64.1 ± 5.4	64.6 ± 4.8	65.6 ± 5.2	0.0125*
TRO	971.8 ± 123.2	892.8 ± 154.1	833.3 ± 128.6	<0.0001*	52.3 ± 4.4	52.4 ± 4.2	52.1 ± 4.8	0.7940	70.3 ± 6.9	70.0 ± 7.0	70.7 ± 7.1	0.5438

**Note:** TH: slice thicknesses (mm); LFC: lateral femoral condyle, LTP: lateral tibial plateau, MFC: medial femoral condyle, MTP: medial tibial plateau, PAT: patella, TRO: trochlea. Statistical Analysis: repeated-measures ANOVA.

**Table 4:** Results of the paired samples t-tests or paired Wilcoxon signed rank test.

	T1		T2		PD	
	TH4 *versus* TH3	TH5 *versus* TH3	TH4 *versus* TH3	TH5 *versus* TH3	TH4 *versus* TH3	TH5 *versus* TH3
LFC	<0.0001*^#^	<0.0001*^#^	<0.0001*^#^	0.0039*	0.1029	0.0004*^#^
LTP	<0.0001*^#^	<0.0001*^#^	0.7420	0.0905	0.0014*	<0.0001*^#^
MFC	<0.0001*^#^	<0.0001*^#^	0.8896	0.4973	0.0221*	0.0240*
MTP	<0.0001* ^# a^	<0.0001*^ # a^	0.9337	0.1240	0.0733	0.0460
PAT	<0.0001*^#^	<0.0001*^#^	0.2520	0.1926	0.3640	0.0070*
TRO	0.0004*^#^	<0.0001*^#^	0.8422	0.6719	0.6964	0.5328

**Note:** TH: slice thicknesses (mm); LFC: lateral femoral condyle, LTP: lateral tibial plateau, MFC: medial femoral condyle, MTP: medial tibial plateau, PAT: patella, TRO: trochlea. Statistical Analysis: data normally distributed: paired t-test (^a^: data non-normally distributed: paired Wilcoxon signed rank test). * Significant results under the original correction (comparisons limited to slice thicknesses only, *P* < 0.025). ^#^ Results that remained significant under the comprehensive Bonferroni correction (18 comparisons in total, *P* < 0.0014).

## Data Availability

The data and material underlying this article are available in the article.

## LIST OF ABBREVIATIONS

MRI: Magnetic Resonance Imaging
MAGIC: Magnetic Resonance Image Compilation
LFC: Lateral Femoral Condyle
LTP: Lateral Tibial Plateau
MFC: Medial Femoral Condyle
MTP: Medial Tibial Plateau
PAT: Patella
TRO: Trochlea
FLAIR: Fluid Attenuated Inversion Recovery
STIR: Short-Tau Inversion Recovery
PSIR: Phase Sensitive Inversion Recovery
DIR: Double Inversion Recovery
MDME: Multi-Dynamic Multi-Echo
ROI: Region Of Interest
ICC: Intraclass Correlation Coefficient
SD: Standard Deviation
ANOVA: Analysis Of Variance

## STANDARDS OF REPORTING

STROBE guidelines were followed.
